# Beyond Neuroinflammation: Targeting the Microbe–Barrier–Brain Axis Through Precision Nutrition in Neurological Long COVID (Neuro-PASC)

**DOI:** 10.3390/nu18203420

**Published:** 2026-10-18

**Authors:** Amador Velázquez De Castro-Bono, Gracia Castro-Luna, José Luis Guil-Guerrero

**Affiliations:** 1 Hospital Central de La Defensa “Gómez Ulla”, 28047 Madrid, Spain; avelazquezcb@gmail.com; 2 Department of Nursing, Physiotherapy and Medicine, University of Almería, 04120 Almería, Spain; graciacl@ual.es; 3 Food Technology Division, University of Almería, 04120 Almería, Spain

**Keywords:** neuro-PASC, long COVID, precision nutrition, microbe–barrier–brain axis, intestinal permeability, microbiome dysbiosis, neuroinflammation, biomarkers, personalised medicine, multi-omics

## Abstract

Post-acute Neurological Sequelae of SARS-CoV-2 infection (Neuro-PASC) contribute substantially to Long COVID-related disability, with cognitive impairment, chronic fatigue, dysautonomia, and neuropsychiatric symptoms among its most debilitating manifestations. Although its pathogenesis remains incompletely understood, evidence implicates persistent immune activation, intestinal barrier dysfunction, microbiota dysbiosis, microbiome-derived metabolic alterations, mitochondrial dysfunction, and neuroimmune interactions in sustaining neuroinflammation and neurological impairment. This narrative review examines precision nutrition as a potential pathway-oriented strategy for Neuro-PASC. We synthesise evidence linking the gut microbiota, intestinal permeability, immunometabolic pathways, mitochondrial function, and neurological outcomes, while evaluating nutritional interventions targeting these mechanisms. We focus on candidate biomarkers, including serum zonulin, fatty acid-binding protein 2 (FABP2), faecal short-chain fatty acids (SCFAs), and the kynurenine-to-tryptophan ratio, which may support patient stratification and personalised nutritional strategies. We also propose a stepwise framework involving dietary stabilisation, functional foods, targeted supplementation, and microbiome-based interventions, alongside practical considerations for post-exertional symptom exacerbation, dysautonomia, and mast cell-related symptoms. Finally, we identify priorities for future research, including multi-omics integration, artificial intelligence, digital health, and biomarker-stratified randomised controlled trials. Because current evidence remains predominantly observational and mechanistic, precision nutrition should be considered a complementary strategy requiring rigorous clinical validation.

## 1. Introduction

### 1.1. Neurological Long COVID (Neuro-PASC): Clinical Burden and Under-Addressed Treatment Needs

Post-acute neurological sequelae of SARS-CoV-2 infection (Neuro-PASC) are characterised by cognitive impairment, severe fatigue, autonomic nervous system dysfunction, sleep disturbances, and neuropsychiatric symptoms, persisting even several months following acute illness [1]. Neuro-PASC can adversely affect daily functioning, occupational capacity, and quality of life. Nonetheless, the lack of standardised criteria and tools for Neuro-PASC diagnosis, reliable outcome measurements, and reproducible biomarkers makes patient stratification and inter-study comparisons difficult [2].

Long-term cognitive deficits have been reported in various longitudinal studies among patients [3]; however, the symptoms and their courses vary widely across individuals. This variability suggests that symptoms may reflect both persistent biological processes and potentially reversible changes. Heterogeneous mechanisms underlie Neuro-PASC, necessitating phenotype definitions that account for both biology and symptoms.

### 1.2. Evolution of Long COVID Paradigms: Shift Toward Systemic and Barrier-Associated Theories

Early models of Long COVID focused on neuroinflammation as the key pathway responsible for persistent neurological symptoms. While the neurocentric models contributed greatly to understanding the vulnerability of the central nervous system after SARS-CoV-2 infection and have initiated important mechanistic research, recent studies have identified persistent immune activation, endothelial dysfunction, coagulation disorders, autonomic dysfunctions, and changes in the composition of the gut microbiome as further mechanisms of disease pathogenesis [4].

A growing body of data confirms that peripheral biological processes interact closely with those in the central nervous system rather than operating independently. Endothelial dysfunction, impaired intestinal permeability, and gut dysbiosis have been linked to the development of persistent neurological symptoms, although causal relationships have not been fully established [5]. This suggests a systemic view in which bidirectional interactions among immune, vascular, intestinal, and nervous pathways can drive neuroinflammation. Further research is needed to determine whether barrier dysfunction is a primary pathological process or a secondary consequence.

A vascular and endothelial hypothesis has also gained prominence as a complementary mechanism. Fibrin-rich microclots resistant to physiological fibrinolysis, together with persistent endothelial dysfunction, have been proposed to impair cerebral microcirculation and contribute to cognitive symptoms independently of, or alongside, gut-derived mechanisms. Direct evidence of blood–brain barrier disruption accompanied by sustained systemic inflammation has been documented in individuals with long COVID-associated cognitive impairment, reinforcing endotheliopathy as a mechanism warranting further integration with barrier- and microbiome-focused models such as the one proposed in this review [6].

### 1.3. Reason for a Nutritional Approach: Nutritional Approach as the Intersection Between Diet, Microbiome, and Neuroinflammation

Nutrition is a potentially modifiable factor that affects immune regulation, microbial communities, and metabolic stability. While pharmacological therapy is necessary to control SARS-CoV-2 infection and some manifestations of Long COVID, immunomodulatory drugs may not be appropriate for all patients because of insufficient evidence, side effects, and the condition’s heterogeneity [7].

Diet has been found to affect gut microbial diversity, microbial metabolite production, and intestinal barrier properties [8]. Consumption of foods rich in fibre, polyphenols, unsaturated fatty acids, and fermented products can stimulate microbial pathways that support improved epithelial function and reduce systemic inflammation. However, interventions vary, and evidence that diet directly affects nervous system recovery remains limited. Therefore, nutrition can be recommended as a complementary tool rather than a separate treatment.

### 1.4. Main Hypothesis and Objectives

This literature review hypothesises that gut dysbiosis and barrier dysfunction are critical elements of the pathological process in Neuro-PASC. Available data demonstrate associations among altered microbial communities, systemic immunity, permeability, and neurological symptoms. The directionality of such associations remains unclear [9].

Rather than treating dysbiosis as an independent cause, we consider it one component of a potentially bidirectional microbe–barrier–brain axis interaction. Dysbiosis, barrier dysfunction, systemic inflammation, metabolic alterations, and neurological abnormalities may influence one another, but their temporal and causal relationships remain unresolved.

Against this background, we review microbial composition and function, barrier function, immune communication, and neurology. We evaluate the quality of evidence in each case, unresolved questions, and directions for future work.

### 1.5. Positioning of This Review Within Current Literature

Several recent publications address related territory and merit distinction. A scoping review following PRISMA-ScR and Joanna Briggs Institute (JBI) methodology systematically mapped 50 studies on diet and nutrition across the full spectrum of Long COVID symptoms, providing a rigorous, broad-based synthesis of the intervention and observational literature [10]. Separately, a narrative review examined precision nutrition and gut–brain axis modulation as a strategy for preventing neurodegenerative diseases such as Alzheimer’s disease, Parkinson’s disease, and multiple sclerosis [11].

This review differs from both in scope and intended contribution. Rather than addressing Long COVID broadly or precision nutrition across neurodegenerative diseases, it focuses specifically on Neuro-PASC and develops a hypothesis-generating biomarker–intervention framework that links candidate biomarkers, clinical phenotypes, biological pathways, and nutritional intervention hypotheses. The framework is intended to identify testable relationships for future biomarker-stratified clinical trials rather than to provide validated treatment recommendations. This narrative synthesis uses a structured literature search and evidence-characterisation approach, but it is not a systematic review and does not provide a formal risk-of-bias assessment comparable to a systematic review or meta-analysis. Readers seeking a systematically mapped evidence base for Long COVID nutrition more broadly should consult the scoping review by Bigman et al. [10].

Although many of the biological mechanisms discussed in this review are shared with other disorders involving the gut–brain axis, their relevance to Neuro-PASC requires consideration within the specific clinical and biological context of post-acute SARS-CoV-2 infection. This review integrates neurological phenotyping, intestinal barrier and microbiome alterations, immunometabolic pathways, candidate biomarkers, and nutritional strategies within a Neuro-PASC-specific translational framework. The purpose is not to propose that these mechanisms are unique to Neuro-PASC, but to determine how far current evidence supports their integration within neurological Long COVID and to identify testable gaps between mechanistic observations and nutritional intervention.

## 2. Methods

### 2.1. Literature Search Strategy

A targeted literature search was conducted to identify publications relevant to the microbe–barrier–brain axis and precision nutrition in neurological Long COVID (Neuro-PASC). We searched five bibliographic platforms: PubMed, Scopus, Web of Science, ScienceDirect, and SpringerLink. We used Google Scholar as a supplementary source to identify additional relevant publications.

The search strategy covered four thematic areas: (1) Neuro-PASC and Long COVID; (2) neurological manifestations, including neurocognitive symptoms, dysautonomia, neuroinflammation, and fatigue; (3) the gut–brain axis, microbiome alterations, intestinal barrier function, and microbial metabolites; and (4) nutritional and microbiome-targeted interventions, including dietary approaches, probiotics, prebiotics, synbiotics, postbiotics, and polyphenols. We combined search terms using Boolean operators and adapted them to each platform’s syntax. The exact search strings, thematic focus, search dates, and retrieved-record counts are provided in Supplementary Table S1.

The search aimed to identify literature relevant to the review objectives rather than establish a comprehensive, systematically screened corpus. Additional publications were identified through supplementary Google Scholar searches and examination of relevant reference lists. The methodological framework drew on established approaches to narrative synthesis and transparent evidence mapping.

The methodological framework drew on established approaches to narrative synthesis and principles of transparent evidence mapping [12,13].

We conducted the final literature search in May 2026, covering records indexed from database inception to the final search date.

### 2.2. Literature Selection and Narrative Synthesis

We selected literature relevant to the review’s central objective: potential interactions among the intestinal microbiome, barrier function, microbial metabolites, and neurological manifestations of Long COVID, and the possible contribution of nutritional strategies. Priority was given to studies directly addressing Long COVID or post-COVID-19 conditions, particularly those investigating neurological or neurocognitive manifestations.

Where direct evidence in Neuro-PASC was limited, studies from broader Long COVID populations, other clinical settings, and experimental models were considered when they provided relevant mechanistic context. We interpreted their findings based on their relevance and limitations, without assuming that results from other populations or models necessarily applied to Neuro-PASC.

The evidence was integrated narratively, without quantitative pooling. Interpretation considered the consistency and directness of findings, their biological plausibility, and their relevance to the review objectives. We distinguished observational associations, experimental findings, and clinical intervention results to avoid equating proposed mechanisms with demonstrated clinical effects.

### 2.3. Inclusion and Exclusion Criteria

We considered peer-reviewed scientific articles, authoritative reviews, and other relevant scientific sources addressing Neuro-PASC, Long COVID, or mechanisms potentially relevant to neurological post-acute sequelae. Eligible publications provided information in at least one of the following domains:

1. Clinical characterisation: cognitive dysfunction, fatigue, post-exertional symptom exacerbation, dysautonomia, sleep disturbances, or neuropsychiatric manifestations of Long COVID.
2. Microbe–barrier–brain mechanisms: gut microbiome dysbiosis, intestinal permeability, epithelial barrier dysfunction, microbial translocation, systemic inflammation, neuroimmune signalling, or blood–brain barrier alterations.
3. Immunometabolic pathways: tryptophan–kynurenine metabolism, SCFAs, microbial metabolites, neurotransmitter pathways, oxidative stress, mitochondrial dysfunction, and brain energy metabolism.
4. Nutritional interventions: dietary patterns, dietary fibre, resistant starch, omega-3 fatty acids, polyphenols, micronutrients, functional foods, and other nutritional approaches relevant to the proposed mechanisms.
5. Microbiome-targeted interventions: probiotics, prebiotics, synbiotics, postbiotics, fermented foods, next-generation probiotics, and faecal microbiota transplantation.
6. Precision nutrition and clinical translation: biomarkers, phenotyping, multi-omics approaches, digital health, artificial intelligence, and biomarker-stratified clinical trials.

We excluded studies focused exclusively on acute COVID-19 without relevance to post-acute neurological manifestations, unrelated nutritional outcomes, or insufficient information to support the interpretation of relevant findings.

Human studies were prioritised when available. Experimental and mechanistic studies were included when they helped explain potentially relevant biological pathways. We selectively considered evidence from other inflammatory, metabolic, or neurological conditions and interpreted it as indirect evidence rather than proof of equivalent effects in Neuro-PASC.

We did not impose a uniform intervention, comparator, and outcome structure because the review encompassed heterogeneous observational, mechanistic, experimental, and interventional research.

### 2.4. Data Extraction and Synthesis

We extracted information on study design, population, neurological phenotype, intervention or exposure, biological pathways, microbiome and barrier findings, biomarkers, nutritional components, clinical outcomes, and reported limitations. For intervention studies, we additionally considered intervention type, duration, proposed biological targets, relevant microbial or metabolic outcomes, and safety or tolerability. For biomarker studies, we recorded the biological compartment, marker investigated, association with disease phenotype, and potential clinical relevance.

Not all studies provided information across every category. Missing information was recorded as not reported, without inference. Extracted data were checked against the original sources, with particular attention to distinguishing reported findings from interpretations arising from cross-study synthesis.

We did not apply formal study-quality scoring or quantitative evidence ranking because the literature varied substantially in design, population, intervention, outcomes, and methodological purpose. Instead, we considered study design, relevance to Neuro-PASC, outcome assessment, and principal methodological limitations. These included small sample sizes, heterogeneous clinical phenotypes, cross-sectional designs, variable microbiome methods, inconsistent biomarker definitions, limited longitudinal evidence, and the scarcity of adequately powered randomised controlled trials.

We presented nutritional interventions lacking Neuro-PASC-specific clinical evidence as candidates for investigation rather than established treatments. We present extracted information in the main text and tables by evidence domain, preserving distinctions between mechanistic plausibility, observational associations, and demonstrated clinical effects.

## 3. Pathophysiology of the Microbe–Barrier–Brain Axis in Neuro-PASC

The mechanisms discussed in this section are not necessarily specific to Neuro-PASC. Human Long COVID studies provide the primary disease-specific evidence, while experimental findings and evidence from other inflammatory, gastrointestinal, metabolic, neurological, or post-viral conditions provide mechanistic context. Do not interpret the latter as evidence that the same mechanisms or nutritional responses have been demonstrated in Neuro-PASC.

### 3.1. Gastrointestinal Viral Material Persistence and Intestinal Barrier Dysfunction

Post-mortem examinations and gastrointestinal biopsies indicate that SARS-CoV-2 RNA and viral proteins may persist in extrapulmonary tissues for months after acute infection. SARS-CoV-2 RNA and nucleocapsid protein have been detected in gastrointestinal mucosal tissues, including intestinal epithelium, although evidence for persistent replication-competent virus remains limited [4,14]. In one biopsy study of patients with inflammatory bowel disease, SARS-CoV-2 RNA was detected in gut mucosa approximately seven months after infection, while viral nucleocapsid protein persisted in intestinal tissue [14]. These findings support the possibility of persistent gastrointestinal viral material but do not establish an active viral reservoir in all patients with Long COVID [14].

Persistent viral RNA or proteins may contribute to prolonged immune activation, but the causal relationship between tissue persistence and Long COVID symptoms remains unresolved [14,15]. SARS-CoV-2 infection can affect intestinal epithelial integrity, and experimental models have demonstrated disruption of tight-junction proteins and impaired barrier function following infection [14]. ACE2 is highly expressed in intestinal epithelial cells and participates in intestinal physiological processes beyond viral entry. Alteration of ACE2-related signalling may consequently affect epithelial function and intestinal homeostasis [16].

Impaired intestinal barrier function may facilitate the passage of microbial products into circulation, potentially contributing to systemic immune activation [17]. Clinical and experimental evidence supports an association between SARS-CoV-2 infection, intestinal barrier disruption, and microbial translocation, although the magnitude and persistence of these changes vary among studies [17].

Thus, gastrointestinal viral persistence and barrier dysfunction represent plausible components of the microbe–barrier–brain axis, but their specific contribution to neurological Long COVID remains uncertain. Biomarkers that identify persistent gastrointestinal viral material, epithelial injury, and microbial translocation could help determine whether these processes define clinically relevant Neuro-PASC phenotypes. Prospective longitudinal studies are needed to establish their temporal relationship with neurological symptoms and determine whether nutritional or microbiome-targeted interventions can modify them [4].

### 3.2. Microbiome Dysbiosis and Tryptophan Metabolism Dysregulations

Studies have consistently reported changes in microbiota composition and metabolism in patients with Long COVID [8,9]. Specifically, studies have repeatedly reported decreases in the abundance of SCFA-producing microorganisms, along with increases in potentially pro-inflammatory microorganisms. Several studies report similar patterns, although methodological differences in populations, sampling, sequencing, and analytical approaches limit direct comparison.

Microbiome dysbiosis can affect tryptophan metabolism through prolonged immune stimulation. Inflamed tissue releases inflammatory cytokines, which increase indoleamine 2,3-dioxygenase activity and promote tryptophan catabolism through the kynurenine pathway rather than serotonin synthesis [18]. A recent meta-analysis demonstrated consistently higher kynurenine-to-tryptophan ratios in patients with Long COVID than in healthy controls [19]. These findings strengthen the association between persistent immune activation and dysregulated tryptophan metabolism. Nevertheless, current evidence remains largely observational and does not establish causality.

Elevated kynurenine production could reduce available serotonin and produce metabolites that affect glutamatergic neurotransmission and neuroinflammation [18]. Such mechanisms may contribute to neurological symptoms such as fatigue, cognitive problems, disrupted sleep cycles, and mood disorders. However, the relative contribution of these mechanisms may vary by clinical characteristics and disease stage.

Existing evidence provides a biological rationale for investigating nutrition-based interventions. A diet that supports microbial biodiversity, along with selected prebiotics, probiotics, and fermentable fibres, may help restore normal microbial metabolic function. Clinical trials are still needed to confirm the neurological impact of such approaches on Neuro-PASC patients.

### 3.3. Neuroimmunological Communication and Microglial Activation

The gut–brain axis is an important communication pathway linking the intestinal microbiota and the central nervous system through neurological, immune, endocrine, and metabolic processes. Among those mechanisms, the vagus nerve is considered the primary neural pathway for bidirectional signalling between the gut and the brain. According to recent research, SARS-CoV-2 infection can impair vagus nerve function, thereby attenuating the cholinergic anti-inflammatory reflex and inducing sustained immune activation [20].

Disrupted neural signalling may alter physiological processes related to systemic inflammation and contribute to neuro-immune activation. Persistent peripheral immune activation can activate microglia through cytokines, damage-associated molecular patterns, and microbiome-derived metabolites, potentially affecting CNS function.

Microglia play an essential role in maintaining neuronal homeostasis, synaptic remodelling, and host defence. Acute microglial activation may therefore be protective during viral infection. However, prolonged activation may promote chronic neuroinflammation, disrupt synaptic plasticity, and impair neural network function [21]. These alterations may contribute to cognitive dysfunction, fatigue, and neuropsychiatric manifestations reported in patients with Neuro-PASC.

Current evidence supports persistent neuroimmune activation in Long COVID, although the precise contribution of vagal dysfunction remains uncertain [22]. Most available data originate from experimental models or indirect clinical observations rather than direct measurements of vagal integrity or microglial activity in humans [23]. Consequently, the hypothesis that persistent vagal neuropathy drives Neuro-PASC remains biologically plausible but needs further validation.

These observations nevertheless identify several promising therapeutic opportunities. Noninvasive vagus nerve stimulation has attracted growing interest because it can modulate autonomic balance and attenuate systemic inflammation. Preliminary clinical studies suggest favourable effects on autonomic symptoms [24], although evidence specific to neurological Long COVID remains limited. Future longitudinal studies integrating autonomic testing, advanced neuroimaging, circulating inflammatory biomarkers, and microbiome profiling will be essential for clarifying the temporal relationship among vagal dysfunction, microglial activation, and neurological recovery.

**Figure 1:**
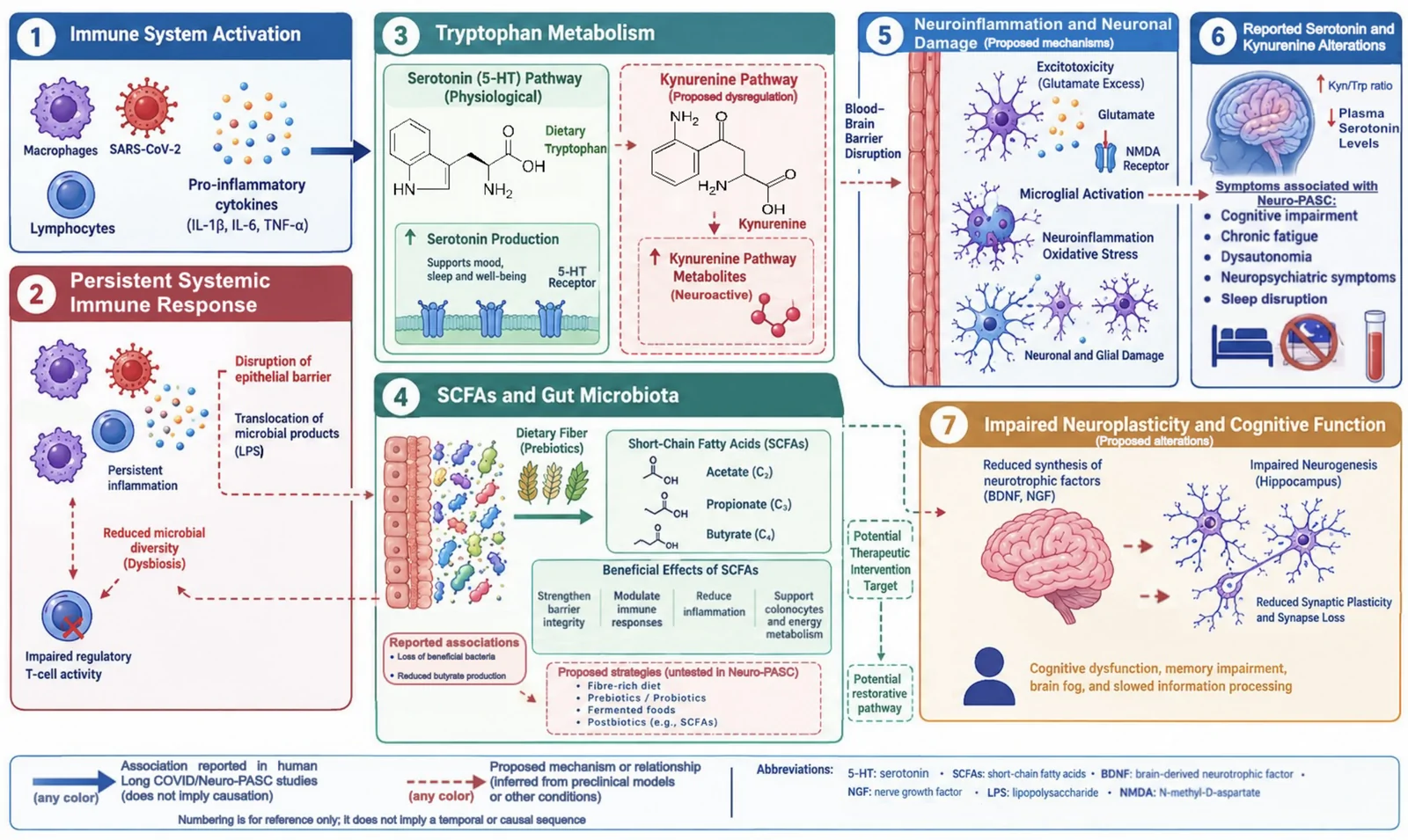
Proposed microbe–barrier–brain axis in neurological Long COVID (Neuro-PASC): a hypothesis-generating model. Panel numbers are for reference only and do not imply a temporal or causal sequence. Solid arrows indicate associations reported in human Long COVID/Neuro-PASC studies and do not imply causation; dashed arrows indicate proposed mechanisms or relationships inferred from preclinical models, acute COVID-19, or other conditions, and are not established in Neuro-PASC. Arrow colour is for visual grouping only. (**1**) Immune activation with elevated pro-inflammatory cytokines. (**2**) Persistent systemic inflammation, epithelial barrier disruption, and microbial translocation have been reported in Long COVID; their directionality and their relationship with dysbiosis and regulatory T-cell function remain unresolved. (**3**) Inflammatory signalling may divert tryptophan metabolism from the serotonin pathway towards the kynurenine pathway; an elevated kynurenine-to-tryptophan ratio has been reported in Long COVID, whereas the neuroactive effects of kynurenine-pathway metabolites remain hypothetical. (**4**) Reduced faecal and circulating SCFAs, particularly butyrate, have been reported in Long COVID; the fermentation of dietary fibre to SCFAs is a general physiological process, and the nutritional and microbiome-targeted strategies shown are proposed and untested in Neuro-PASC. (**5**) Blood–brain barrier disruption has been documented in individuals with Long COVID and cognitive impairment; excitotoxicity, microglial activation, and oxidative stress are proposed mechanisms, supported mainly by experimental or indirect evidence. (**6**) Altered kynurenine-to-tryptophan ratio and reduced serotonin have been reported in some Long COVID cohorts; the listed symptoms are clinical features of Neuro-PASC, and their relationship with these alterations has not been established. (**7**) Altered neuroplasticity is proposed as a contributor to cognitive symptoms.

Figure 1 summarises the proposed interactions between gut dysbiosis, vagal dysfunction, systemic immune activation, and microglial activation within the microbe–barrier–brain axis, illustrating how these interconnected pathways may contribute to persistent neurological symptoms in Long COVID.

### 3.4. Mitochondrial Dysfunction and Brain Energy Metabolism

There is increasing research that implicates the role of mitochondrial dysfunction in the continued neurological symptoms experienced by Long COVID patients. Research has shown that people with Long COVID experience impaired oxidative phosphorylation, decreased ATP synthesis, and excessive production of reactive oxygen species [25]. This can impair energy production in high-energy-demand tissues, such as the central nervous system.

Systemic inflammation and oxidative stress can also exacerbate the problems caused by mitochondrial dysfunction. Oxidative and nitrosative species damage mitochondrial membranes, mitochondrial enzymes, and mitochondrial DNA [25]. This reduces energy production efficiency and increases oxidative stress. Research also indicates that proteins of SARS-CoV-2 interact with mitochondrial pathways in the host cell [26], although the persistence and clinical relevance of these interactions after acute infection remain uncertain.

Mitochondrial dysfunction could impact cerebral energy metabolism. Imaging studies using magnetic resonance spectroscopy and positron emission tomography have shown that there is metabolic dysfunction and hypometabolism in patients with Neuro-PASC [25,27]. In chronic neuroinflammation, metabolism may shift from high-energy aerobic pathways to low-energy anaerobic pathways. Lactate buildup can occur, leading to an energy shortage that may impair neuronal function. Metabolic disturbances may contribute to brain fog, slowed cognition, and continued fatigue.

In addition, mitochondrial dysfunction could contribute to neuroinflammation through the release of mtDAMPs, and oxidative stress, immune system activation, and cellular bioenergetic dysfunction create a vicious circle [25]. This hypothesis is biologically plausible, but direct evidence in Neuro-PASC patients remains limited.

Metabolic imaging advances have provided more evidence for impaired brain bioenergetics [27]. However, these approaches are largely limited to research because they are scarce and expensive. Similarly, generalisation of muscle metabolic results to the CNS is not justified due to tissue-specific differences in metabolic adaptation [28].

Hence, nutritional strategies targeting mitochondrial function represent a potential area for investigation. Nutritional interventions that increase mitochondrial metabolic efficiency and reduce systemic inflammation and oxidative stress may increase cellular energy generation. Finally, ketogenic therapies and metabolic supplements might prove useful because they provide an alternative energy source for the brain when glucose metabolism is impaired, in the form of ketones [29]. Despite a plausible mechanistic rationale, adequately powered randomised controlled trials are needed to determine whether these mitochondrial-targeting nutritional strategies provide clinically meaningful benefits in Neuro-PASC.

Several nutritional compounds have been proposed as mitochondrial-targeting strategies in post-viral fatigue syndromes. Coenzyme Q10 participates in mitochondrial electron transport and also has antioxidant properties [30], whereas alpha-lipoic acid contributes to redox regulation and mitochondrial enzyme activity [31]. L-carnitine transports long-chain fatty acids into mitochondria, providing a mechanistic rationale for investigating it in disorders associated with impaired energy metabolism. NAD+ precursors, including nicotinamide riboside, may support cellular redox reactions and mitochondrial metabolism [32]. Evidence for these interventions remains heterogeneous and is derived mainly from post-viral fatigue syndromes, observational studies, or conditions other than Neuro-PASC. A recent randomised trial of nicotinamide riboside in Long COVID increased circulating NAD+ concentrations but did not demonstrate significant improvements in cognition or fatigue [33]. These findings support further investigation but do not establish mitochondrial supplementation as an effective treatment for Neuro-PASC.

### 3.5. Alterations in Immunometabolic Pathways: Tryptophan–Kynurenine Metabolism, SCFAs, and Neurotransmitter Balance

Persistent immune activation significantly affects host metabolism and likely contributes to neurological impairment through several interlinked immunometabolic mechanisms. Tryptophan metabolism and short-chain fatty acid (SCFA) production have been studied closely because they are directly linked to the gut microbiota and neuroimmune regulation [34,35].

Pro-inflammatory cytokines upregulate the enzyme indoleamine 2,3-dioxygenase (IDO), which affects tryptophan metabolism, leading to its conversion from serotonin to the kynurenine pathway [18]. This process can increase kynurenine production and may reduce serotonin availability. Various kynurenine pathway metabolites are neuroactive substances that can affect glutamatergic transmission, oxidative stress, and neuroinflammation. An increased kynurenine-to-tryptophan ratio has been identified in patients with Long COVID through meta-analyses [19].

These metabolic changes offer a biological rationale for fatigue, cognitive dysfunction, sleep disorders, and neuropsychiatric signs [19,36]. Nevertheless, current evidence remains largely associative, and direct causal relationships have not been established. It is also unclear whether altered tryptophan metabolism represents a primary pathogenic mechanism or reflects persistent systemic inflammation. Longitudinal studies that integrate metabolomics, immune profiling, and clinical outcomes are therefore needed before kynurenine metabolites can be adopted as reliable biomarkers or therapeutic targets.

Gut microbial dysbiosis may further amplify neurological dysfunction by reducing SCFA production, particularly butyrate. These microbial metabolites support intestinal barrier integrity, regulate immune tolerance, and influence microglial maturation through multiple signalling pathways [37]. These experimental studies also suggest that SCFAs promote neurotrophic signalling, preserve synaptic plasticity, and modulate neuroinflammatory responses. Reduced SCFA production may therefore contribute to persistent immune activation and impaired neuronal function.

Although restoring SCFA synthesis represents a potential therapeutic hypothesis for neurological Long COVID, the clinical relevance of SCFA alterations and the most appropriate intervention remain uncertain. Current data suggest that increasing fermentable fibre intake and supporting endogenous microbial fermentation through a prebiotic-rich diet and other microbiome-directed nutritional strategies may increase endogenous SCFA production. Such dietary strategies warrant investigation alongside, rather than in preference to, direct postbiotic approaches. However, evidence on the clinical effectiveness of oral SCFA supplementation in Neuro-PASC remains insufficient, and controlled clinical studies are needed to evaluate its potential benefits [38].

In summary, the gut microbiota’s production of tryptophan and SCFAs provides insight into how gut dysbiosis affects neurological function through both metabolic and immune pathways. Nevertheless, with further research, clinicians can effectively modulate these pathways in patients with neurological Long COVID.

## 4. Nutrition-Based Interventions

Evidence supporting the nutritional approaches discussed in this section varies substantially in directness to Neuro-PASC. Most available evidence concerns biological mechanisms, broader Long COVID populations, or other inflammatory and gastrointestinal conditions. These findings provide mechanistic context but do not establish clinical efficacy in neurological Long COVID.

### 4.1. Enhancement of Intestinal Barrier Function by Nutrition

Systemic inflammation can affect the intestinal barrier by disrupting tight junctions between epithelial cells and increasing permeability. Some studies have found elevated levels of biomarkers of intestinal barrier dysfunction among people with Long COVID; however, the data are heterogeneous, and assay standardisation is needed [39]. Higher permeability may facilitate microbial translocation and contribute to sustained immune activation and neurological symptoms.

These observations support nutritional interventions that enhance epithelial barrier function. Sufficient protein intake, along with nutrients such as glutamine, zinc, and other bioactive compounds, may improve epithelial healing and mucosal stability [39]. This approach has shown potential in experimental and clinical studies of other gastrointestinal conditions; however, there is no direct evidence related to neurologic Long COVID [40]. Thus, current evidence is insufficient to support barrier-targeted nutritional interventions as Neuro-PASC-specific treatments.

### 4.2. Omega-3 Polyunsaturated Fatty Acids and Specialised Pro-Resolution Lipid Mediators

Persistent inflammation in Long COVID has increased research interest in omega-3 polyunsaturated fatty acids, as these substances act as precursors of specialised pro-resolving lipid mediators that promote the resolution of inflammation [41]. Some patients showed abnormalities in the lipid mediator profile and endothelial dysfunction, suggesting impaired resolution of inflammation rather than its persistence [41].

These findings increase the biological plausibility of omega-3 supplementation’s potential benefits. Preclinical studies suggest anti-inflammatory, neuroprotective, and endothelial protective effects, while clinical trials are in their early stages [42]. The optimal dosage of the substance, its form, period of administration, and population to which it should be applied is yet to be determined.

### 4.3. Dietary Fibre, Resistant Starch and SCFA Production

Gut dysbiosis may reduce microbial fermentation of dietary fibre and, consequently, decrease SCFA production, particularly butyrate [43]. Low faecal SCFA levels have been associated with increased intestinal permeability, immune dysregulation, and alterations in the gut–brain axis [44]. Several studies have reported reduced faecal butyrate levels in patients with post-viral symptoms [45]. However, the relationship between SCFA concentrations and neurological symptoms remains unclear.

SCFAs may also link dietary interventions to vagal signalling. Dietary fibre fermentation produces microbial metabolites that can influence intestinal epithelial and enteroendocrine cells, which communicate with vagal afferents through hormonal and neural pathways. Enteroendocrine cells can release mediators such as serotonin, GLP-1, CCK, and PYY in response to luminal nutrients and microbial signals, providing plausible routes for gut-to-brain communication. These mechanisms suggest that dietary fibre and microbiome-directed interventions could indirectly influence autonomic and neuroimmune signalling through the vagus nerve. This mechanism should thus be considered a hypothesis requiring clinical investigation [46].

Increasing dietary fibre intake may support endogenous SCFA synthesis by providing fermentable substrates for intestinal bacteria [36]. Resistant starch and fermentable fibres can favour the growth of specific microbial populations and the production of their metabolites [44]. However, individuals with gastrointestinal symptoms may tolerate rapid increases in fibre poorly. Nutritional interventions should therefore introduce fermentable substrates gradually and account for individual gastrointestinal tolerance when modifying dietary composition. Whether a personalised approach guided by microbiome analysis provides greater benefits remains unclear.

### 4.4. Polyphenols and Flavonoids: Microbial Biotransformation and Neuroprotection

Polyphenols from many foods undergo extensive microbial biotransformation before entering the systemic circulation, forming compounds with biological properties distinct from those of the parent molecules [47]. Based on experimental evidence, these compounds can exert antioxidant activity, modulate inflammatory signalling pathways, and affect neuronal physiology [48]. Several observational studies have found correlations between high polyphenol consumption and improved cognitive outcomes; however, no causal relationship has been established [49].

These findings support the possibility that polyphenol-rich diets may contribute to gut–brain axis homeostasis. Yet, individual variability in polyphenol metabolism by microbiota complicates the predictability of responses to both foods and dietary supplements [50]. Currently, evidence remains insufficient to support specific polyphenol supplementation strategies, whereas polyphenol-rich dietary patterns may offer a broader and more physiologically integrated source of these compounds.

### 4.5. Key Micronutrients (Vitamin D, B-Complex, Magnesium, Zinc, Selenium) in Neuroimmune Modulation

Alterations in magnesium and electrolyte status have been reported in Long COVID, although their relationship with neurological symptoms and clinical outcomes remains uncertain [51]. A lack of important micronutrients may impair enzymatic processes involved in CNS control [52]. This deficiency may disrupt ATP production in human mitochondrial cells. Evaluating intracellular micronutrient status could be a useful step in future personalised nutritional protocols, although validated thresholds specific to Neuro-PASC are lacking. Recent epidemiological research indicates that severe vitamin D deficiency increases the risk of long-term post-viral cognitive dysfunction [52]. Current evidence does not support multivitamin therapy as a substitute for targeted corrections of neurological disorders during Long COVID. Restoring adequate micronutrient status may support biochemical processes relevant to neurological function, although this remains hypothetical in the context of Neuro-PASC.

## 5. Microbiome-Targeted Therapeutic Strategies

The microbe–barrier–brain axis has been proposed as a conceptual framework for understanding interactions among gut, immune, metabolic, and neurological processes in Long COVID. This framework provides a rationale for investigating microbiome-targeted nutritional interventions, but it does not establish that microbiome dysfunction initiates neurological symptoms. Potential therapeutic approaches include probiotics, prebiotics, synbiotics, postbiotics, fermented foods, and faecal microbiota transplant (FMT). Despite promising results regarding their mechanisms and preliminary clinical studies, robust evidence from adequately powered randomised controlled trials remains limited [53]. As such, microbiome-based therapeutic modalities should currently be considered investigational approaches rather than approved therapies.

**Figure 2:**
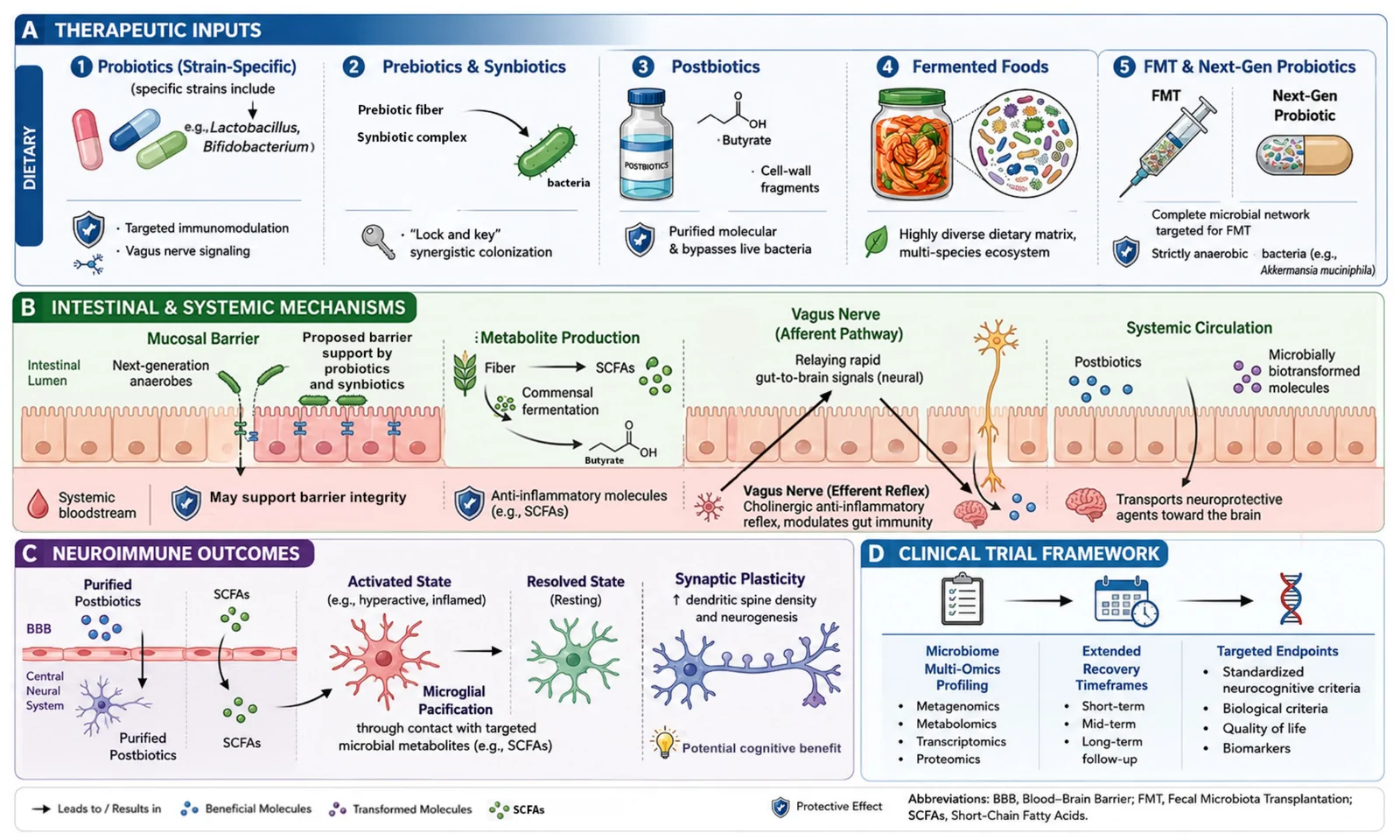
Strategic framework for microbiome-targeted therapeutics in Neuro-PASC. (**A**) Diverse interventional strategies encompass strain-specific probiotics, precision prebiotics/synbiotics, direct postbiotic administration, complex fermented dietary matrices, and advanced faecal microbiota transplantation (FMT). (**B**) These interventions may influence intestinal barrier function, vagal signalling, and SCFAs production. (**C**) Systemically available microbial metabolites may influence blood–brain barrier function, microglial activity, and hippocampal synaptic plasticity through several proposed gut–brain signalling pathways. (**D**) Clinical validation of these biological mechanisms requires strictly controlled, adequately powered longitudinal trials using advanced multi-omics baseline testing and standardised, objective neurocognitive reporting criteria.

Figure 2 summarises the primary microbiome-based therapy strategies and their mechanisms of action.

### 5.1. Probiotics: Strain-Specific Evidence for Neurological Long COVID

Probiotics have become an area of interest because certain bacterial strains can influence immune regulation, gut permeability, and the gut–brain axis [54]. Experiments indicate that some *Lactobacillus* and *Bifidobacterium* strains can reduce inflammatory cytokine production, strengthen epithelial function, and regulate vagal nerve signalling. These findings provide biological plausibility for potential effects of probiotics on pathways relevant to neurological symptoms, but they do not establish neurological efficacy in Neuro-PASC.

However, clinical data are currently scarce. Studies currently have small sample sizes, sample heterogeneity, varying formulations, and differing administration times. As a result, it is hard to compare outcomes across studies and draw firm conclusions. It is worth noting that there are no class effects of probiotics; their effects are strain-specific [55].

Thus, future clinical trials should focus on identifying the most efficacious strains, determining the optimal treatment duration, and using microbiome sequencing and metabolomic analysis.

### 5.2. Prebiotics and Synbiotics

Prebiotics selectively promote microbial growth by supplying fermentable substrates that support bacterial metabolism. Synbiotics combine probiotics with complementary prebiotics to enhance bacterial survival, colonisation, and metabolism. These methods aim to enhance microbial diversity and the production of beneficial metabolites, mainly SCFAs.

There is much mechanistic evidence for prebiotics; however, little clinical evidence exists for their use in Long COVID with neurologic manifestations [55]. There is substantial variation in the components, dosages, and bacterial viability among commercially available synbiotics, making comparisons difficult [55]. In addition, it remains unknown whether current preparations contain sufficient fermentable substrates to meaningfully alter the microbiome.

Gastrointestinal manifestations may limit tolerance to rapidly increasing amounts of fermentable fibres. Thus, a gradual increase and personalised dietary advice appear more suitable than standardisation. Future clinical trials need to assess synbiotic efficacy using biomarkers.

### 5.3. Postbiotics and Microbial Metabolites

Postbiotics are active ingredients produced by microbes, including SCFAs, microbial peptides, cell wall components, and metabolites that can affect host physiology independently of viable organisms. This approach is gaining popularity because its effectiveness does not depend on colonisation success.

Lower levels of SCFAs in both circulation and stool samples were observed in Long COVID, indicating reduced microbial metabolic activity [37,54]. Evidence suggests that butyrate and its metabolites enhance epithelial barrier integrity, modulate regulatory T-cell activity, and reduce neuroinflammation [56]. These mechanisms provide a rationale for investigating SCFAs and other postbiotics as candidate interventions in Neuro-PASC.

However, clinical applications remain in their early stages. Further studies are needed on the pharmacodynamics, dosing, formulation, and safety of purified postbiotics. It is also unknown whether metabolites retain the biological properties of the indigenous microflora’s metabolic pathways. Consequently, researchers should compare purified postbiotics with dietary methods that stimulate endogenous metabolite production.

### 5.4. Fermented Foods and Diet-Induced Modulation of the Microbiota

Fermented foods are thought to expose individuals to a combination of live microorganisms, their metabolites, and bioactive substances that may affect gut microbial diversity and the immune system. Regular intake of fermented foods was associated with increased microbial diversity and lower inflammatory marker levels; however, causality is difficult to establish [57].

Compared with probiotic supplements, fermented foods introduce the human body to a wider range of microbial communities and their metabolites through fermentation [55]. Yet substantial differences in microbial composition arise from variations in production processes, storage conditions, and pasteurisation. Moreover, microbial content varies significantly between commercial products.

Fermented foods can be considered components of a healthy diet rather than standalone therapies. Clinical trials are needed to investigate the beneficial effect of specific fermented foods in Long COVID.

### 5.5. Innovative Treatment Approaches: Second-Generation Probiotics and Faecal Microbiota Transplants (FMT)

Progress in understanding the microbiome has spurred interest in next-generation probiotics based on anaerobic commensals with specific metabolic capabilities [58]. Some of these candidate microorganisms have demonstrated protective effects on the barrier and immune systems in preclinical experiments, but human data are lacking.

Another novel therapeutic approach proposed for severely disrupted microbiomes is FMT. Some preliminary reports of positive outcomes in certain patients with post-viral syndrome symptoms have recently emerged; however, these findings are based solely on uncontrolled observations and small studies [45]. This means there is still considerable uncertainty about its efficacy, durability, indications, and mechanisms of action.

Finally, safety should also be mentioned. FMT requires thorough donor screening and standardisation of its manufacturing process to avoid the spread of infections and any unwanted biological impacts [53]. Currently, FMT use should be limited to clinical trials.

### 5.6. Critical Review of Ongoing and Completed Clinical Trials (2024–2026)

Recent clinical trials evaluating the efficacy of microbiome-based treatment approaches have yielded promising results, though with significant inconsistencies [59]. Variations in study populations, intervention procedures, baseline microbiota states, outcome measures, and follow-up periods make cross-study comparisons difficult.

Several clinical trials have reported effects of selected probiotic or synbiotic therapies on fatigue, gastrointestinal symptoms, or cognitive outcomes in post-viral or Long COVID populations [45,59]. However, the extent to which these findings apply specifically to neurological Long COVID remains uncertain.

Another drawback is the high degree of patient heterogeneity. Recent studies include patients with highly diverse symptom profiles, with no differentiation by neurological involvement, gastrointestinal disorders, inflammation, or initial microbial state. This may explain inconsistent therapeutic outcomes and low statistical power.

Future clinical trials should use standardised diagnostic criteria, neurological outcome measures, longitudinal multi-omics assessment, and patient stratification by biomarkers. Future multicenter randomised trials will be needed to evaluate the clinical efficacy of microbiome treatments.

## 6. Dietary Patterns for Neuro-PASC

Dietary interventions offer a useful means to alter several biological pathways implicated in Neurological Long COVID. Unlike single-nutrient interventions, dietary patterns can influence several biological pathways simultaneously, including gut microbiota composition, microbial metabolite production, immune signalling, oxidative stress, and endothelial function. However, evidence that these effects translate into clinical benefits in Neuro-PASC remains limited. Dietary patterns with established effects on these pathways may provide a useful basis for hypothesis generation within multimodal Neuro-PASC research. Table 1 summarises dietary approaches investigated or proposed for selected post-viral, neurological, gastrointestinal, and autonomic symptoms relevant to Long COVID.

These approaches are derived partly from evidence in conditions other than Neuro-PASC and should not be interpreted as validated treatments for neurological Long COVID.

### 6.1. The Mediterranean Diet as a Microbiome-Modulating Dietary Pattern

The Mediterranean diet has been extensively studied in relation to microbial diversity and systemic inflammation [63,64]. Frequent consumption of vegetables, fruits, legumes, whole-grain products, nuts, and extra-virgin olive oil provides significant amounts of dietary fibre, polyphenols, and unsaturated fatty acids, thereby supporting the colonisation of beneficial microorganisms, including butyrate-producing bacteria such as *Faecalibacterium prausnitzii*. Adherence to such a dietary pattern is associated with increased microbial diversity and lower levels of circulating inflammatory markers, such as C-reactive protein and interleukin-6 [65].

The Mediterranean diet typically contains fewer highly processed foods, excessive sugar intake, and food additives than Western-style dietary patterns [65]. Extra virgin olive oil contains monounsaturated fatty acids and phenolic compounds that exhibit anti-inflammatory and endothelium-protective properties [66]. Despite promising biological mechanisms and strong biological plausibility, no direct clinical data show the benefits of this diet in Neuro-PASC patients. However, it remains one of the more extensively studied dietary patterns for individuals with chronic inflammation [65].

### 6.2. Mechanisms of Dietary Anti-Inflammatory and Neuroprotective Action

Certain food constituents may affect biological processes involved in neuroinflammation and neuronal recovery. The long-chain polyunsaturated omega-3 fatty acids, such as eicosapentaenoic acid (EPA) and docosahexaenoic acid (DHA), reduce the release of pro-inflammatory mediators and increase the production of specialised pro-resolving lipid mediators that promote the resolution of inflammation without merely suppressing immunity [41].

Microorganisms first metabolise polyphenols in berries, leafy green vegetables, dark chocolate, tea, and other foods into antioxidants and immunomodulators. Experimental and observational evidence suggests that some polyphenols may influence neurotrophic and inflammatory signalling, including pathways involving BDNF. However, the clinical relevance of these effects in Neuro-PASC remains uncertain [50]. Other bioactive compounds, such as sulforaphane and luteolin, have shown neuroprotective effects in experimental models involving endogenous antioxidant and cellular stress-response pathways [67,68].

Limited research exists on the clinical use of these compounds in patients with Long COVID. Therefore, their importance should be considered only within the context of a healthy dietary pattern.

### 6.3. Practical Dietary Adjustments for Patients with Cognitive Impairment and Fatigue

Fatigue, cognitive decline, and exacerbations of symptoms after physical effort have been reported to impair food preparation and diet in people suffering from Long COVID. Nutrition advice should therefore reflect both metabolic needs and the functional challenges the disease poses.

Approaches can involve minimising the effort required for food preparation through batch cooking, prepared nutritious foods, or meal replacements where appropriate. More often, smaller meals may help preserve energy and reduce post-meal fatigue, although no evidence supports this approach in Long COVID patients. People with parosmia, dysgeusia, or hypersensitivity might benefit from individualised dietary adjustments to facilitate food intake without compromising nutritional status [69].

Effective nutrition depends on both diet composition and the feasibility and adherence to it. This is why individualised diet counselling might increase nutritional intake alongside fluctuations in functional capacity in Neuro-PASC.

### 6.4. Nutritional Management of Histamine Intolerance, Mast Cell Activation Syndrome (MCAS), and Postural Orthostatic Tachycardia Syndrome (POTS)

Some people suffering from Long COVID can have a collection of symptoms associated with histamine intolerance, Mast Cell Activation Syndrome (MCAS), or autonomic dysfunction. Although the relationship between these conditions and Neuro-PASC remains poorly understood, these individuals may benefit from an individualised nutritional approach.

Those patients who have signs of histamine intolerance or MCAS can experience an improvement when following a diet low in histamines for some time, but only under strict medical supervision [70,71]. Because long-term diets are likely to negatively affect food quality, they should be implemented systematically. In particular, this may include gradually reintroducing foods after finishing a low-histamine diet. Reduced diamine oxidase activity has been considered a possible explanation, but its relationship to Neuro-PASC needs to be studied [40].

Increasing fluid and sodium intake and having small meals to reduce splanchnic blood pooling after eating are other recommendations for patients with Postural Orthostatic Tachycardia Syndrome (POTS) or other dysautonomias [61]. Although these dietary recommendations are supported by evidence from POTS populations, their effectiveness in Long COVID has not yet been established. Therefore, it is reasonable to individualise diet plans based on a patient’s cardiovascular health and symptoms.

Targeted dietary intervention in patients with concurrent histamine intolerance, mast cell activation syndrome, and dysautonomia is summarised in Table 1. Overall, this example demonstrates the need for personalised nutrition therapy that considers both symptoms and an individual patient’s nutritional status.

## 7. A Translational Precision Nutrition Framework

### 7.1. Biomarkers of the Microbe–Barrier–Brain Axis

Implementing a precision nutrition approach for Neuro-PASC may require objective evaluation of candidate components of the microbe–barrier–brain axis using biomarkers that require clinical validation. Despite the lack of a biomarker for clinical diagnosis due to insufficient sensitivity and specificity, several candidate biomarkers may provide complementary information about the intestinal barrier, microbial metabolism, and immune activation [72].

Candidate biomarkers may help characterise biological processes relevant to Neuro-PASC and inform hypotheses for future personalised nutritional research. However, the evidence supporting these relationships varies in its directness, ranging from findings in Long COVID populations to studies in other clinical settings and mechanistic investigations. Table 2 summarises selected studies underpinning the proposed biomarker–intervention framework, highlighting their principal findings, the strategies discussed, and their relevance and limitations for Neuro-PASC. These associations should not be interpreted as evidence that a biomarker can currently predict treatment response.

Several limitations constrain the clinical interpretation of these candidate biomarkers. Serum zonulin measurements require caution because some commercial assays lack adequate analyte specificity [72]. FABP2 may indicate enterocyte injury, but it is not specific to Neuro-PASC [72]. Faecal SCFA concentrations reflect the balance between microbial production, intestinal absorption, host metabolism, and faecal transit. They do not directly quantify microbial production or host exposure [56]. Faecal calprotectin indicates intestinal inflammation but is not specific to Long COVID, as it may also be elevated in other gastrointestinal diseases [82]. The kynurenine-to-tryptophan (Kyn/Trp) ratio may reflect altered tryptophan metabolism, but it does not uniquely establish IDO-1 activity [18,19,80]. These markers may help investigate relevant biological processes; however, prospective Neuro-PASC studies still need to establish their analytical validity, clinical utility, and ability to predict nutritional treatment responses.

### 7.2. Neuro-PASC Clinical Phenotyping for Personalised Nutrition

Given the substantial clinical heterogeneity observed in Long COVID, a standard nutritional approach is unlikely to benefit all affected individuals [83]. Patient stratification based on clinical presentation and biological abnormalities may help guide future personalised strategies, although the proposed phenotypes require validation.

The following phenotypes are proposed research constructs rather than validated Neuro-PASC endotypes. They combine clinical features with candidate biological markers to illustrate hypotheses that future stratified studies could test. These categories are not mutually exclusive, and individual patients may present features of several phenotypes.

The dysbiotic/gastrointestinal phenotype includes bloating, food intolerance, gastrointestinal symptoms, and potentially altered faecal SCFA profiles. Microbiome-targeted nutritional interventions and the gradual introduction of fermentable fibres warrant investigation [43,44].

The hyperinflammatory/neurovascular phenotype encompasses cognitive impairment and may involve elevated inflammatory markers or an altered kynurenine-to-tryptophan (Kyn/Trp) ratio. Dietary strategies involving omega-3 fatty acids, polyphenols, and other nutrients with potential immunomodulatory effects could be investigated in this context [41,48].

The mast cell activation/histamine phenotype includes flushing, food intolerance, postprandial symptom worsening, and allergy-like manifestations. A low-histamine diet may warrant investigation in selected cases, although its efficacy for this proposed Neuro-PASC phenotype remains uncertain [60].

The autonomic/dysautonomic phenotype includes orthostatic intolerance, tachycardia, and exercise intolerance, sometimes accompanied by volume depletion. Nutritional approaches, including individualised fluid and sodium intake, may be considered alongside established clinical management, according to the patient’s clinical status and contraindications [61,62].

Limitations of biomarker-guided nutritional strategies. Interpret candidate biomarkers associated with these phenotypes as potential indicators of biological processes, not as validated tools for selecting nutritional interventions. Serum zonulin measurements require particular caution because some widely used commercial immunoassays may not specifically detect pre-haptoglobin-2, the protein identified as zonulin. Such results cannot independently establish intestinal permeability or barrier dysfunction. FABP2 may indicate enterocyte injury but is not specific to Long COVID, and its interpretation depends on assay characteristics and clinical context. Faecal SCFA concentrations are also difficult to interpret because they reflect the balance between microbial production, intestinal absorption, host metabolism, intestinal transit, and sample handling. Moreover, an association between a biomarker and a clinical feature does not demonstrate that the biomarker predicts treatment response. Prospective studies are needed to establish the analytical validity, clinical relevance, and predictive utility of these candidate markers in Neuro-PASC.

Despite substantial overlap among these symptom clusters, they may provide a useful framework for investigating personalised nutrition in future studies. Their clinical utility will depend on validating the phenotypes and demonstrating that biomarker-informed interventions improve outcomes compared with approaches that do not use biomarker-based stratification.

### 7.3. A Hypothesis-Generating Biomarker–Intervention Framework

One possible application of mechanism-driven research is to examine whether candidate biomarkers associate with distinct nutritional intervention hypotheses in Neuro-PASC. However, the described algorithm (Table 3) is currently a hypothesis-generating framework rather than a clinically validated decision tool, as validation studies have not yet been conducted.

Table 3 outlines candidate biomarkers, their proposed biological interpretation, and nutritional interventions to evaluate in future clinical studies.

This framework is hypothesis-generating and is not a validated diagnostic or therapeutic algorithm. Evidence categories refer to the support for the proposed biomarker–intervention relationship, not merely to whether the biomarker or intervention has been studied independently. Associations with biological processes do not establish that a biomarker predicts treatment response. No listed biomarker has established predictive validity for nutritional treatment selection in Neuro-PASC. Microbial production, intestinal absorption, host metabolism, transit, and sample handling influence faecal SCFA concentrations. Serum zonulin measurements require caution because some commercial assays may not specifically detect pre-haptoglobin-2.

### 7.4. Stepwise Nutritional Intervention Strategy

**Figure 3:**
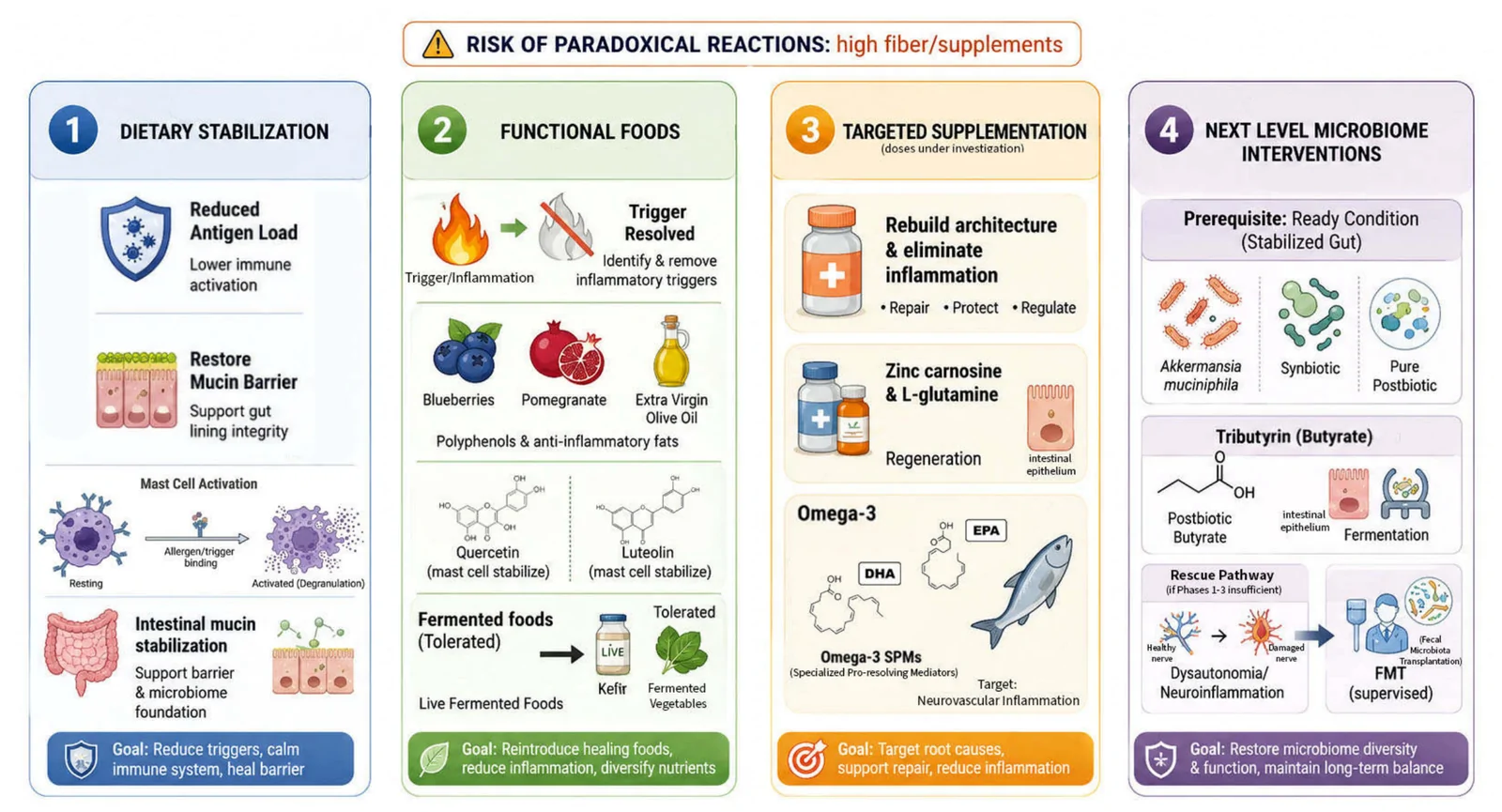
Proposed four investigational nutritional domainsfor restoring gut barrier function and microbiome homeostasis in neurological Long COVID. (**1**) Dietary Stabilisation. This aims to assess whether dietary stabilisation using an individualised Mediterranean-like pattern is tolerated and may influence relevant symptoms and biological pathways. Avoid ultra-processed foods, refined sugar, and certain additives; attempt a low-histamine diet if mast cell activation is suspected. (**2**) Functional Foods. After gastrointestinal symptoms stabilise, gradually incorporate polyphenol-rich foods such as berries, pomegranate, and extra virgin olive oil. Individuals without histamine intolerance may gradually consume some live fermented foods to increase microbiota diversity, while monitoring for any possible worsening of symptoms. (**3**) Targeted Supplementation. Depending on each patient’s clinical and biochemical profile, selected nutritional supplements to promote intestinal mucosal integrity and control inflammation may be introduced. Possible candidates include L-glutamine, zinc-carnosine, and omega-3 fatty acids, although trials have not yet confirmed their specific benefits in Neuro-PASC. (**4**) Investigational Microbiome-Targeted Interventions. In research settings, patients with persistent abnormalities despite dietary intervention could be evaluated for microbiome-targeted approaches, such as precision synbiotics, next-generation probiotics, or postbiotics. Faecal microbiota transplantation should remain a research tool and be used only in controlled clinical trials or select clinical settings until more robust evidence emerges.

Because patients with Neuro-PASC often have multisystem symptoms, autonomic instability, and variable tolerance to dietary interventions, a gradual, phased framework may help structure investigations into nutritional strategies (Figure 3). Rather than rapidly introducing multiple supplements or large increases in dietary fibre, a gradual approach may improve tolerability, although controlled studies have not established its effects on symptom exacerbation. Nevertheless, this proposed framework has not been formally evaluated in randomised clinical trials and should not be interpreted as a validated clinical protocol.

Figure 3 presents a proposed four nutritional frameworks for investigating strategies targeting intestinal barrier function and microbiome-related pathways. These domains may be investigated independently or in combination, depending on the research question and clinical context. The four-domain nutritional framework is intended to organise candidate strategies for future investigation, not to prescribe a sequential therapeutic protocol. Current evidence does not establish that all domains are necessary, that they should be implemented in a particular order, or that this arrangement is superior to alternative approaches.

The domains may overlap and could be investigated individually or in combination, subject to appropriate clinical evaluation and supporting evidence. Similarly, the proposed clinical phenotypes represent conceptual patterns rather than mutually exclusive categories. Individuals with Neuro-PASC may present overlapping features, and their expression may vary over time. Prospective studies are needed to determine whether these domains can provide clinically useful stratification or individualised intervention.

### 7.5. Practical Considerations: Managing Fatigue, Dysautonomia, and Executive Dysfunction

Effective nutritional therapy also requires awareness of the functional barriers presented by many people living with Neuro-PASC. Severe fatigue, symptoms following exertion, cognitive dysfunction, and executive dysfunction can all impair an individual’s capacity to plan meals, prepare food, and follow dietary regimens [81,84].

Thus, dietary advice needs to consider both feasibility and quality. Effort-saving techniques such as batch cooking, nutrient-rich freeze-dried foods, ready-made vegetables, and simplified meal planning may help people reduce physical effort while still ensuring adequate nutrient intake [69]. People with dysautonomia may benefit from frequent smaller meals with lower amounts of simple carbohydrates, helping them avoid postprandial hypotension and splanchnic venous pooling [61,62]. In cases of extreme fatigue when food preparation is very difficult or impossible, complete liquid meals may be an alternative to ensure sufficient energy and micronutrient intake [69].

In sum, personalised dietary interventions should be incorporated into the multidisciplinary approach, considering the dynamic course of the disease and considerable functional disability of people suffering from Neuro-PASC.

## 8. Future Perspectives

Despite advances in understanding the neurophysiological consequences of Long COVID, significant gaps remain, preventing the translation of biological discoveries into evidence-based nutritional interventions. Current evidence points to the microbe–barrier–brain axis, but most available scientific data are observational, cross-sectional, or preclinical, hindering causal inference. Consequently, the principal translational challenge is not the absence of plausible biological mechanisms, but the lack of evidence connecting these mechanisms to reproducible neurological phenotypes and clinically meaningful responses to nutritional intervention. Future studies should establish these links prospectively rather than assume that modulating one component of the axis will improve neurological outcomes. Therefore, researchers have not yet identified validated biomarkers to identify patients who could benefit from nutritional or microbiome-based treatments.

Further investigation will likely integrate systems biology, microbiome sciences, nutritional epidemiology, computational medicine, and clinical phenotyping. These methodologies may improve understanding of disease heterogeneity, identify clinically relevant therapeutic targets, and support the development of personalised nutritional interventions. Beyond uniform dietary guidelines, future interventions will likely target microbial, metabolic, immunological, and clinical endotypes.

**Figure 4:**
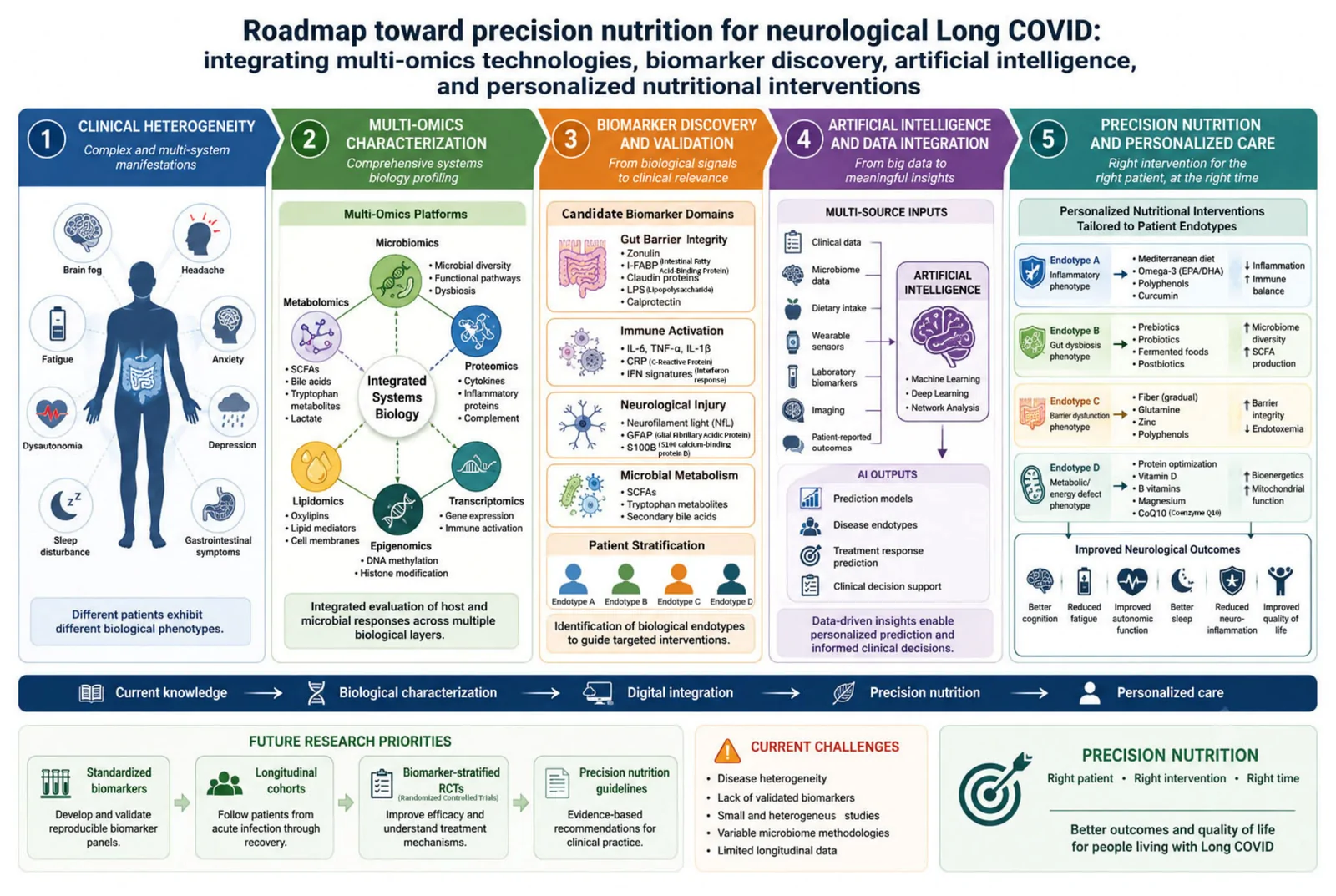
Roadmap toward precision nutrition for neurological Long COVID. It illustrates the proposed framework for developing a personalised nutrition approach for the management of Neuro-PASC by defining five inter-related steps: (**1**) description of clinical and biological heterogeneity in Neuro-PASC; (**2**) use of multi-omics approaches to define molecular disease signatures; (**3**) validation of biomarkers for patient stratification; (**4**) use of artificial intelligence for multidimensional dataset integration; and (**5**) implementation of personalised nutritional interventions based on biological endotypes. The lower part of the figure discusses existing challenges, including the lack of validated biomarkers, a heterogeneous patient population, and a lack of interventional studies.

Figure 4 presents a roadmap of translational steps connecting biological characterisations, biomarker discovery, artificial intelligence, and precision nutrition for neurological Long COVID.

### 8.1. Precision Nutrition in Neurological Long COVID Through Multi-Omics Methods

Multi-omics approaches are crucial for describing the biological complexity of neuroinflammation in Long COVID. Each individual omics approach provides complementary insights that cannot be obtained through traditional clinical research [85].

Microbiomics allows characterisation of microbiota composition and its functional properties related to chronic dysbiosis. Metabolomics helps identify biologically active microbial metabolites, including SCFAs, bile acids, and tryptophan derivatives involved in neuroimmune interactions. Bile acids deserve particular attention because microbial conversion of primary to secondary bile acids generates signalling molecules that interact with receptors involved in metabolic and immune regulation. Experimental and emerging clinical evidence suggests that altered bile-acid signalling may influence intestinal barrier function, systemic inflammation, blood–brain barrier integrity, and glial responses. However, the relevance of these pathways to Neuro-PASC remains insufficiently defined [86]. Transcriptomics, proteomics, lipidomics, and epigenomics help characterise host responses, including immune activation, endothelial dysfunction, mitochondrial metabolism, oxidative stress, and tissue repair [85].

Future longitudinal studies should integrate bile-acid profiling with microbiome composition, intestinal permeability markers, inflammatory mediators, and neurological outcomes to determine whether bile-acid signatures can contribute to patient stratification or nutritional intervention design.

Combining these data could help identify biologically distinct disease endotypes based on the condition’s underlying mechanisms rather than symptoms, enabling more specific nutritional recommendations. Different endotypes can include patients suffering from chronic intestinal barrier disruption, inflammation, mitochondrial damage, autonomic disorders, and metabolic dysfunction.

Despite their high potential, clinical applications of multi-omics approaches are limited by high cost, technical complexity, a lack of standardised analytical pipelines, and limited availability of longitudinal datasets. Validation in other independent cohorts is required for clinical use of multi-omics approaches [85].

### 8.2. Artificial Intelligence and Digital Nutrition in Long COVID

Artificial intelligence emerged as a potential tool for addressing biologically heterogeneous conditions, such as Long COVID. Machine learning can process multidimensional datasets comprising information on clinical features, nutrient intake, microbiome profiles, biochemical markers, wearable device data, and subjective patient reports to discover patterns that are difficult to detect with traditional statistical methods [87,88].

Digital nutrition systems can further support personal diet management by continuously monitoring nutrient adherence, gastrointestinal symptoms, sleep quality, physical activity, autonomic measurements, and symptom dynamics. Combining these longitudinal data with microbial and metabolomic assessments may support adaptive dietary strategies during the recovery period [69].

Nevertheless, most artificial intelligence models are still in early development and require validation before practical application.

### 8.3. Designing Biomarker-Stratified Randomised Controlled Trials

Currently, most randomised controlled trials have included clinically heterogeneous patients with Long COVID, without biomarker stratification. This may reduce the impact of nutritional intervention because these patients’ pathophysiology is highly heterogeneous.

Future randomised controlled trials should consider biomarker stratification before initiating nutritional intervention. Appropriate biomarkers include inflammatory cytokines, intestinal permeability biomarkers, microbial metabolites, neurofilament light chain, glial fibrillary acidic protein, autonomic biomarkers, mitochondrial metabolites, and microbiome analysis.

This approach could reduce biological heterogeneity and allow future trials to test whether specific biomarker-defined subgroups respond differently to particular nutritional interventions.

In addition to subjective patient reports, future trials should consider objective neurological endpoints. These could include neuropsychological testing, autonomic function testing, neuroimaging, metabolomics, and microbiome sequencing.

Table 4 summarises the principal research priorities required to advance precision nutrition for neurological Long COVID.

### 8.4. Testing the Microbe–Barrier–Brain Axis Hypothesis: Key Scientific Objectives

Growing evidence suggests that dysbiosis, intestinal barrier disruption, sustained immune activation, and neurological dysfunction are interrelated in Long COVID syndrome. However, it is unclear whether these factors are causative, downstream effects, or adaptive mechanisms.

Longitudinal studies must concurrently assess changes in intestinal permeability, microbiome composition, immune activation, metabolomics, autonomic dysfunction, and neurologic outcomes over the course of the disease. These studies will clarify the temporal relationships among these phenomena and help identify potential treatment opportunities.

Clinical trials are required to determine whether restoring intestinal barrier integrity and modulating the microbiome alleviate neurologic symptoms. Integrating nutritional intervention with microbiome modulation may be more beneficial than either method alone, although further research is required.

Multicentre collaborations may be necessary to achieve sufficient statistical power and replicability. Standardising diagnostic criteria, sampling methods, laboratory techniques, and outcome measures should be a key priority for future research. Overall, these studies may help determine whether the microbe–barrier–brain axis represents a clinically relevant and therapeutically modifiable pathway in Neuro-PASC. The axis should currently be regarded as a working hypothesis rather than an established causal pathway. Longitudinal studies with repeated measurements of the microbiome, intestinal barrier, immune and metabolic markers, autonomic function, and neurological outcomes are needed to establish temporal relationships and directionality.

**Figure 5:**
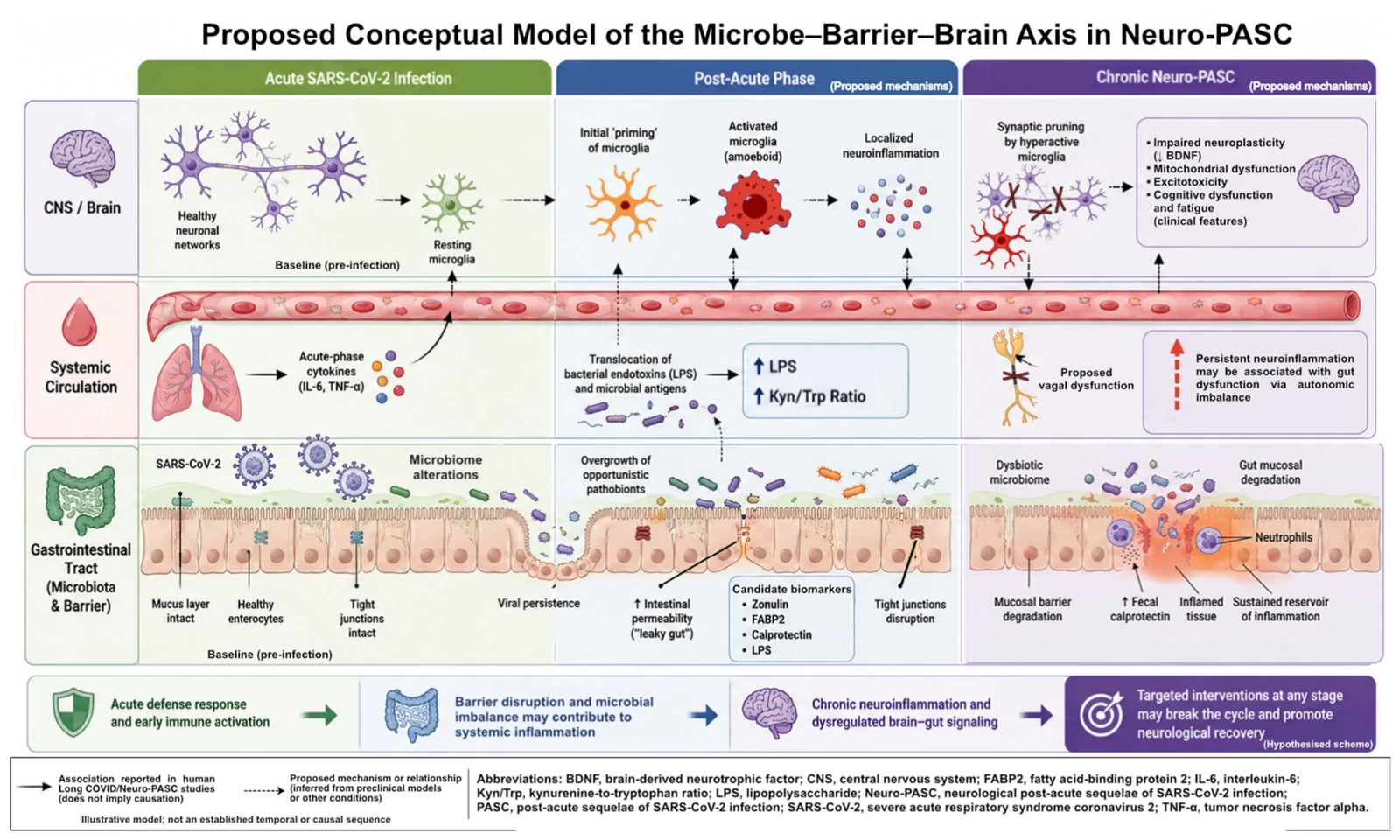
Proposed conceptual model of the microbe–barrier–brain axis in neurological Long COVID (Neuro-PASC). The three panels illustrate processes reported or hypothesised at different phases after SARS-CoV-2 infection. They do not represent an established temporal or causal progression: no study has yet followed these processes longitudinally within the same individuals, and the directionality between gut, systemic, and central nervous system compartments remains unresolved (see Section 8.4). Solid arrows indicate associations reported in human COVID-19/Long COVID studies and do not imply causation; dashed arrows indicate proposed mechanisms or relationships inferred from preclinical models or other conditions. Acute phase: SARS-CoV-2 may infect ACE2-expressing intestinal epithelial cells; gut microbiome alterations and systemic acute-phase cytokines (IL-6, TNF-α) have been reported. Post-acute phase: persistence of viral RNA or antigen in gastrointestinal tissue, dysbiosis, increased intestinal permeability, and microbial translocation have been reported; an elevated Kyn/Trp ratio has been described in Long COVID; zonulin, FABP2, faecal calprotectin, and LPS are candidate biomarkers of barrier dysfunction that remain to be validated. Microglial priming and activation are proposed mechanisms supported mainly by experimental or indirect evidence. Chronic phase: persistent dysbiosis, mucosal inflammation, vagal dysfunction, and synaptic and metabolic alterations, including mitochondrial dysfunction, are proposed contributors to neuroinflammation and to the cognitive and fatigue symptoms of Neuro-PASC. The model is hypothesis-generating, and the potential therapeutic targets shown have not been tested in Neuro-PASC.

Figure 5 presents a hypothesis-generating conceptual model of the proposed microbe–barrier–brain axis across the acute, post-acute, and chronic phases of SARS-CoV-2 infection. The phases are illustrative; the model does not represent an established temporal or causal progression and requires testing in the longitudinal studies described above.

### 8.5. Limitations of This Review

Several limitations of this narrative review should be acknowledged. Although we used a structured search strategy, eligibility criteria, and evidence-selection framework, we did not conduct a systematic review or follow PRISMA or PRISMA-ScR reporting requirements. No formal risk-of-bias assessment or quantitative evidence synthesis was performed. Selection and citation bias therefore cannot be excluded. Readers seeking a systematically mapped evidence base for Long COVID nutrition should consult recent scoping reviews of the broader literature [10].

Second, most biomarkers and mechanisms discussed reflect associative evidence from observational studies; almost none have been evaluated in Neuro-PASC-specific randomised controlled trials, and the hypothesis-generating biomarker–intervention framework (Table 2) and the four-phase nutritional protocol (Section 7.4) remain hypothesis-generating conceptual frameworks rather than validated clinical protocols.

Third, several of the nutrient-intervention associations cited throughout this review (for example, zinc carnosine and L-glutamine for intestinal permeability, or curcumin for faecal calprotectin) are derived from populations other than Neuro-PASC, such as exercise-induced permeability or inflammatory bowel disease; their extrapolation to Neuro-PASC, while biologically plausible, has not been directly tested and is flagged as such at each relevant point in the text.

Finally, this is a rapidly evolving field; new biomarker validation studies and clinical trials are likely to alter the evidence base described here within a short time frame, and the framework proposed should be revisited as further data emerge.

## 9. Conclusions

Neurological Post-Acute Sequelae of SARS-CoV-2 infection (Neuro-PASC) is emerging as a complex condition affecting multiple body systems through interactions among the nervous, immune, vascular, and gastrointestinal systems. Recent findings suggest that disruptions in microbe-barrier-brain relationships, including altered microbiome composition, reduced barrier function, sustained immune responses, and impaired neuroimmune communication, are associated with the occurrence and persistence of neurologic disorders following SARS-CoV-2 infection, such as cognitive dysfunction, fatigue, dysautonomia, and others. Although the contribution of each process remains unclear, evidence supports the idea that peripheral mechanisms may contribute to central neurologic dysfunction.

Within this framework, nutritional intervention may influence multiple biological pathways involved in Neuro-PASC. The high degree of clinical and biological variability associated with Long COVID means dietary interventions are unlikely to help all patients. Candidate biomarkers of intestinal permeability, mucosal inflammation, microbial metabolism, and immunometabolic activity may eventually support patient stratification, but their clinical validity and predictive value remain to be established. Current evidence supports investigating structured, stepwise nutrition-based approaches focused on dietary stabilisation, repair of gut barrier integrity, metabolic restoration, and, where needed, microbiome-directed therapy. Although these approaches are biologically plausible and partly supported by evidence from related conditions, randomised clinical trials are still needed to determine their efficacy and safety in Neuro-PASC.

Finally, progress in managing nutritional intervention for Long COVID will depend on shifting the focus from symptomatology to a biologically grounded, mechanism-driven approach. Incorporating advanced omics technologies, biomarker discovery, digital health tracking, and artificial intelligence may help identify disease endotypes and implement precision nutrition therapies. However, important uncertainties remain, and future advancements will require carefully designed biomarker-stratified clinical trials to demonstrate causal effects and identify those most likely to benefit from particular treatments. As the evidence base develops, precision nutrition may become a useful complementary strategy for managing individuals with neurological Long COVID.

## Figures and Tables

**Table 1:** Targeted Dietary Adjustments for Co-occurring Post-Viral Syndromes.

Clinical Condition	Primary Dietary Strategy	Key Micronutrients & Behavioural Adjustments	References
Mast Cell Activation Syndrome (MCAS)/Histamine Intolerance	Strict low-histamine elimination diet; avoidance of aged/fermented foods.	Vitamin C, Vitamin B6, Copper (essential Diamine Oxidase (DAO) enzymatic cofactors).	[60]
Postural Orthostatic Tachycardia Syndrome (POTS)/Dysautonomia	High-sodium and increased-hydration protocols to expand central blood volume.	3–6 g+ sodium daily; 2.5–3 L fluids; frequent, small, low-carbohydrate meals to prevent blood pooling.	[61,62]

**Table 2:** Evidence supporting each candidate biomarker in Long COVID and the indirect evidence for the paired nutritional hypothesis.

Biomarker	Evidence in Long COVID or COVID-19	Paired Intervention	Evidence for the Intervention	Level *	Gap and Limitations for Neuro-PASC
Serum zonulin	Cross-sectional study, 415 participants [73]: plasma zonulin (commercial ELISA) higher in COVID-19 with PASC (median 4.76 mg/mL) than without PASC (3.43 mg/mL) or non-infected controls (3.37 mg/mL); aOR 1.44 (95% CI 1.1–1.9) per unit. In acute COVID-19, zonulin rose with severity and was higher in patients who died [74].	Zinc carnosine, bovine colostrum (L-glutamine, exploratory)	Randomised, double-blind, placebo-controlled crossover trial, 8 healthy volunteers [75]: 14 days of zinc carnosine 37.5 mg and/or colostrum 10 g twice daily reduced the exercise-induced rise in lactulose:rhamnose ratio by ~70%; the combination added an early benefit. Glutamine is discussed in a narrative review of barrier-directed nutrients [39].	L2/L3 (glutamine: L4)	Permeability was measured with a sugar-absorption test in healthy volunteers after strenuous exercise, not with zonulin. One widely used zonulin ELISA does not detect pre-haptoglobin-2 [76], and the kits used in the cited cohorts were not specifically validated. No study has examined whether zonulin identifies patients who respond to these supplements.
Plasma FABP2 (exploratory)	Not elevated in severe acute COVID-19, whereas zonulin and microbial-translocation markers were [74]. Long COVID measurements were not identified.	None proposed	—	L3/—	Findings suggest permeability can increase without enterocyte death, so FABP2 may not reflect barrier dysfunction in COVID-19. It is retained only as an exploratory marker.
Faecal calprotectin	Acute COVID-19, 40 hospitalised patients [77]: calprotectin and IL-6 raised with diarrhoea. Post-acute, 40 patients with diarrhoea and 40 without [78]: 20% remained >200 µg/g at 3 months; 3 had severe ileocolitis on colonoscopy, 2 histologically compatible with Crohn’s disease.	None supported	Curcumin plus piperine in inflammatory bowel disease, randomised placebo-controlled trial, 12 weeks [79]: no change in faecal calprotectin or other inflammatory markers; serum superoxide dismutase increased; heartburn was more frequent.	L2/L3	Persistently raised calprotectin after COVID-19 warrants gastroenterological evaluation rather than nutritional self-treatment. No evidence shows that supplements lower it in this setting.
Reduced faecal butyrate and SCFAs	Prospective cohort, 106 patients [34]: PASC was characterised by lower *F. prausnitzii* and higher *R. gnavus*; neuropsychiatric symptoms and fatigue correlated with nosocomial gut pathogens. Reviews describe depletion of SCFA-producing bacteria [8,9].	Fermentable fibre, resistant starch, prebiotics (within synbiotics)	Randomised, double-blind, placebo-controlled trial, 463 patients [79]: a synbiotic (three *Bifidobacterium* strains plus galacto-oligosaccharides, xylo-oligosaccharides and resistant dextrin) for 6 months increased resolution of fatigue (63% vs. 43%), difficulty concentrating (62% vs. 39%), and memory loss (42% vs. 27%). Mechanistic reviews [43,44].	L2/L1 (synbiotic); L4 (fibre alone)	The benefit cannot be attributed to the prebiotic component; participants were not selected based on faecal butyrate, and the cohort was single-ethnicity. Faecal butyrate reflects production minus absorption, host metabolism and transit. Oral butyrate is not proposed (Section 3.5).
Elevated kynurenine-to-tryptophan (Kyn/Trp) ratio	Systematic review and meta-analysis [19]: higher Kyn/Trp ratio in Long COVID than in controls. Review of serotonin and kynurenine pathway disruption in post-COVID conditions [80].	Vitamin B3 (niacin), polyphenol-rich diets	Narrative review of niacin in neurological disorders other than Long COVID [81]. Meta-analysis of randomised trials in non-Long COVID populations (10 trials, 451 participants): polyphenol supplementation lowered IL-6 and improved verbal memory and executive function [48].	L2/L4 (B3); L3 (polyphenols)	The rationale for vitamin B3 is biochemical (tryptophan is converted to NAD+ through the kynurenine pathway) and untested clinically. The polyphenol trials examined inflammatory and cognitive outcomes, not the Kyn/Trp ratio. The ratio does not uniquely establish IDO-1 activity. No study has linked its value to response to these interventions.

* L1, intervention tested in Long COVID; L2, biomarker or pathway studied in Long COVID or post-acute COVID-19; L3, human studies in acute COVID-19, other diseases, or healthy volunteers; L4, narrative reviews or preclinical evidence. No identified study tested whether a biomarker predicts response to the paired intervention.

**Table 3:** Hypothesis-Generating Biomarker–Intervention Framework for Neuro-PASC.

Candidate Biomarker Profile	Proposed Biological Interpretation	Candidate Nutritional Intervention for Investigation	Evidence Level and Directness	Predictive Validity for Treatment Response	References
Elevated serum zonulin and FABP2	Possible intestinal barrier dysfunction and enterocyte injury. Zonulin assay specificity and the lack of disease specificity of FABP2 limit interpretation.	L-glutamine, zinc carnosine, bovine colostrum.	Indirect evidence. The proposed interventions are supported partly by evidence outside Neuro-PASC. The biomarker–intervention relationship remains unvalidated.	Not established in Neuro-PASC.	[39]
Elevated faecal calprotectin	Persistent intestinal inflammation. Calprotectin is not specific to Long COVID and can be elevated in other gastrointestinal conditions.	Curcumin, *Boswellia serrata*, omega-3 fatty acids.	Indirect evidence. The proposed interventions require Neuro-PASC-specific evaluation.	Not established in Neuro-PASC.	[72,79]
Reduced faecal butyrate	May reflect altered microbial fermentation and SCFA availability. Faecal concentrations do not directly measure microbial production or host exposure.	Resistant starches, targeted prebiotics, and butyrate supplementation	Mechanistic and indirect evidence. The proposed strategies require validation in Neuro-PASC.	Not established in Neuro-PASC.	[8,9,44]
Elevated serum kynurenine-to-tryptophan (Kyn/Trp) ratio	May indicate altered tryptophan metabolism and increased IDO-1 activity. The ratio is not specific to a single inflammatory process.	Polyphenol-rich diets, vitamin B3, microbiome-directed interventions	Mechanistic and indirect evidence. The rationale does not establish clinical efficacy or biomarker-guided benefit in Neuro-PASC.	Not established in Neuro-PASC.	[19,80,81]

**Table 4:** Key priorities for future nutritional research in neurological Long COVID.

Research Domain	Current Limitation	Future Priority	Expected Clinical Impact	References
Multi-omics	Fragmented biological data	Integration of microbiomics, metabolomics, transcriptomics, proteomics, and lipidomics	Biological endotyping	[89]
Biomarkers	Lack of validated diagnostic panels	Development and external validation of standardised biomarker panels	Precision nutrition	[72,87]
Clinical trials	Heterogeneous patient populations	Biomarker-stratified randomised controlled trials	Improved treatment efficacy	[87]
Artificial intelligence	Limited prospective validation	Integration of multimodal clinical, microbiome, and metabolomic datasets	Personalised dietary recommendations	[89,90]
Gut barrier	Limited causal evidence	Longitudinal studies of intestinal barrier function	Targeted barrier-restoration therapies	[39]
Microbiome	Predominantly observational evidence	Mechanistic microbiome intervention trials	Evidence-based microbiome modulation	[8,9,72]
Digital health	Limited nutritional monitoring	Integration of wearable technologies and digital nutrition platforms	Continuous individualised care	[88]
Precision nutrition	Generic dietary recommendations	Endotype-specific nutritional interventions	Optimised neurological recovery	[90]

## Data Availability

No new data were created or analyzed in this study. Data sharing is not applicable to this article.

## Supplementary Materials

The following supporting information can be downloaded at: https://www.mdpi.com/article/10.3390/nu18203420/s1, Table S1: Literature Search Strategy for the Narrative Review on the Microbe–Barrier–Brain Axis and Precision Nutrition in Neurological Long COVID (Neuro-PASC).

## Abbreviations

The following abbreviations are used in this manuscript:

ACE2	Angiotensin-Converting Enzyme 2
aOR	adjusted Odds Ratio
ATP	Adenosine Triphosphate
BDNF	Brain-Derived Neurotrophic Factor
CNS	Central Nervous System
CCK	Cholecystokinin
COVID-19	Coronavirus Disease 2019
DAO	Diamine Oxidase
DHA	Docosahexaenoic Acid
DNA	Deoxyribonucleic Acid
ELISA	Enzyme-Linked Immunosorbent Assay
EPA	Eicosapentaenoic Acid
FABP2	Fatty Acid-Binding Protein 2 (intestinal)
FMT	Faecal Microbiota Transplant
GLP-1	Glucagon-Like Peptide-1
IDO/IDO-1	Indoleamine 2,3-Dioxygenase 1
IL-6	Interleukin-6
JBI	Joanna Briggs Institute
Kyn/Trp	Kynurenine-to-Tryptophan ratio
LPS	Lipopolysaccharide
MCAS	Mast Cell Activation Syndrome
mtDAMPs	Mitochondrial Damage-Associated Molecular Patterns
NAD+	Nicotinamide Adenine Dinucleotide
Neuro-PASC	Neurological Post-Acute Sequelae of SARS-CoV-2 infection
PASC	Post-Acute Sequelae of COVID-19/SARS-CoV-2 infection
POTS	Postural Orthostatic Tachycardia Syndrome
PRISMA	Preferred Reporting Items for Systematic Reviews and Meta-Analyses
PRISMA-ScR	Preferred Reporting Items for Systematic Reviews and Meta-Analyses extension for Scoping Reviews
PYY	Peptyde YY
RNA	Ribonucleic Acid
SARS-CoV-2	Severe Acute Respiratory Syndrome Coronavirus 2
SCFA(s)	Short-Chain Fatty Acid(s)
TNF-α	Tumour Necrosis Factor alpha
