# Supplementary material for: Beyond Neuroinflammation: Targeting the Microbe–Barrier–Brain Axis Through Precision Nutrition in Neurological Long COVID (Neuro-PASC)
Source: Nutrients. 2026 Oct 18;18(20):3420. doi: 10.3390/nu18203420 (PMC13708222; doi:10.3390/nu18203420)
Supplement: Supplementary file 1 [file nutrients-18-03420-s001.zip › nutrients-4581787-supplementary.pdf]

Supplementary Table S1. Literature Search Strategy for the Narrative Review on the Microbe–Barrier–Brain Axis and Precision Nutrition in Neurological Long COVID (Neuro-PASC).

Database	Search date	Search line	Exact search string	Focus area	Results (n)
PubMed	May 26	1	("Long COVID"[Title/Abstract] OR "post-COVID-19 condition"[Title/Abstract] OR "post-acute sequelae of SARS-CoV-2"[Title/Abstract] OR Neuro-PASC[Title/Abstract])	Core Neuro-PASC literature	10,585
PubMed	May 26	2	(("Long COVID"[Title/Abstract] OR "post-COVID-19 condition"[Title/Abstract] OR "post-acute sequelae of SARS-CoV-2"[Title/Abstract] OR Neuro-PASC[Title/Abstract]) AND ("neurological symptoms"[Title/Abstract] OR neurocognitive[Title/Abstract] OR dysautonomia[Title/Abstract] OR neuroinflammation[Title/Abstract] OR fatigue[Title/Abstract]))	Neurological manifestations	3,433
PubMed	May 26	3	(("Long COVID"[Title/Abstract] OR "post-COVID-19 condition"[Title/Abstract] OR "post-acute sequelae of SARS-CoV-2"[Title/Abstract] OR Neuro-PASC[Title/Abstract]) AND ("gut-brain axis"[Title/Abstract] OR microbiome[Title/Abstract] OR microbiota[Title/Abstract] OR dysbiosis[Title/Abstract] OR "intestinal barrier"[Title/Abstract] OR "intestinal permeability"[Title/Abstract] OR "short-chain fatty acids"[Title/Abstract] OR tryptophan[Title/Abstract] OR kynurenine[Title/Abstract] OR "bile acids"[Title/Abstract]))	Microbiome, barrier and metabolites	258

PubMed	May 26	4	(("Long COVID"[Title/Abstract] OR "post-COVID-19 condition"[Title/Abstract] OR "post-acute sequelae of SARS-CoV-2"[Title/Abstract] OR Neuro-PASC[Title/Abstract]) AND (nutrition[Title/Abstract] OR dietary[Title/Abstract] OR probiotic[Title/Abstract] OR prebiotic[Title/Abstract] OR synbiotic[Title/Abstract] OR postbiotic[Title/Abstract] OR polyphenol[Title/Abstract]))	Nutritional interventions	155
Scopus	May 26	1	TITLE-ABS-KEY("Long COVID" OR "post-COVID-19 condition" OR "post-acute sequelae of SARS-CoV-2" OR Neuro-PASC)	Core Neuro-PASC literature	15,709
Scopus	May 26	2	TITLE-ABS-KEY(("Long COVID" OR "post-COVID-19 condition" OR "post-acute sequelae of SARS-CoV-2" OR Neuro-PASC) AND ("neurological symptoms" OR neurocognitive OR dysautonomia OR neuroinflammation OR fatigue))	Neurological manifestations	5,810
Scopus	May 26	3	TITLE-ABS-KEY(("Long COVID" OR "post-COVID-19 condition" OR "post-acute sequelae of SARS-CoV-2" OR Neuro-PASC) AND ("gut-brain axis" OR microbiome OR microbiota OR dysbiosis OR "intestinal barrier" OR "intestinal permeability" OR "short-chain fatty acids" OR tryptophan OR kynurenine OR "bile acids"))	Microbiome, barrier and metabolites	450
Scopus	May 26	4	TITLE-ABS-KEY(("Long COVID" OR "post-COVID-19 condition" OR "post-acute sequelae of SARS-CoV-2" OR Neuro-PASC) AND (nutrition OR dietary OR probiotic OR prebiotic	Nutritional interventions	405

			OR synbiotic OR postbiotic OR polyphenol))		
Web of Science	May 26	1	("Long COVID" OR "post-COVID-19 condition" OR "post-acute sequelae of SARS-CoV-2" OR Neuro-PASC)	Core Neuro-PASC literature	713
Web of Science	May 26	2	("Long COVID" OR Neuro-PASC) AND (neurocognitive OR dysautonomia OR neuroinflammation OR fatigue)	Neurological manifestations	3,175
Web of Science	May 26	3	("Long COVID" OR Neuro-PASC) AND (microbiome OR dysbiosis OR "gut-brain axis" OR "intestinal barrier" OR tryptophan)	Microbiome, barrier and metabolites	221
Web of Science	May 26	4	("Long COVID" OR Neuro-PASC) AND (nutrition OR dietary OR probiotic OR prebiotic OR polyphenol)	Nutritional interventions	170
ScienceDirect	May 26	1	("Long COVID" OR "post-COVID-19 condition" OR "post-acute sequelae of SARS-CoV-2" OR Neuro-PASC)	Core Neuro-PASC literature	8,591
ScienceDirect	May 26	2	("Long COVID" OR Neuro-PASC) AND (neurocognitive OR dysautonomia OR neuroinflammation OR fatigue)	Neurological manifestations	4,394
ScienceDirect	May 26	3	("Long COVID" OR "post-COVID-19 condition" OR Neuro-PASC) AND ("gut-brain axis" OR microbiome OR microbiota OR dysbiosis OR "intestinal barrier" OR "intestinal permeability" OR "short-chain fatty acids" OR tryptophan OR kynurenine OR "bile acids")	Microbiome, barrier and metabolites	876
ScienceDirect	May 26	4	("Long COVID" OR Neuro-PASC) AND (nutrition OR dietary OR probiotic OR prebiotic OR polyphenol)	Nutritional interventions	1,379
SpringerLink	May 26	1	("Long COVID" OR "post-COVID-19 condition" OR "post-acute sequelae of SARS-CoV-2" OR Neuro-PASC)	Core Neuro-PASC literature	7,247

SpringerLink	May 26	2	("Long COVID" OR "post-COVID-19 condition" OR Neuro-PASC) AND ("neurological symptoms" OR neurocognitive OR dysautonomia OR neuroinflammation OR fatigue)	Neurological manifestations	3,987
SpringerLink	May 26	3	("Long COVID" OR "post-COVID-19 condition" OR Neuro-PASC) AND ("gut-brain axis" OR microbiome OR microbiota OR dysbiosis OR "intestinal barrier" OR "intestinal permeability" OR "short-chain fatty acids" OR tryptophan OR kynurenine OR "bile acids")	Microbiome, barrier and metabolites	817
SpringerLink	May 26	4	("Long COVID" OR "post-COVID-19 condition" OR Neuro-PASC) AND (nutrition OR dietary OR probiotic OR prebiotic OR synbiotic OR postbiotic OR polyphenol)	Nutritional interventions	939
Google Scholar	May 26	1	"neurological Long COVID" nutrition microbiome	Neurological Long COVID and nutrition	46
Google Scholar	May 26	2	"Long COVID" "intestinal barrier" microbiota metabolites	Mechanistic and translational evidence	913
Google Scholar	May 26	3	"Long COVID" probiotics prebiotics dietary intervention fatigue cognition	Candidate nutritional interventions	697
